# Non-bacterial thrombotic endocarditis masquerading as infective endocarditis: a paraneoplastic process

**DOI:** 10.1093/jscr/rjaf227

**Published:** 2025-04-15

**Authors:** Ujjawal Kumar, Daniel Sitaranjan, Sambhavi Sneha Kumar, Harry Smith, Fadi Al-Zubaidi, Stephen Large

**Affiliations:** School of Clinical Medicine, University of Cambridge, Hills Road, Cambridge, CB2 0SP, United Kingdom; Department of Cardiac Surgery, Royal Papworth Hospital, Papworth Road, Cambridge Biomedical Campus, Cambridge, CB2 0AY, United Kingdom; Department of Cardiac Surgery, Royal Papworth Hospital, Papworth Road, Cambridge Biomedical Campus, Cambridge, CB2 0AY, United Kingdom; School of Clinical Medicine, University of Cambridge, Hills Road, Cambridge, CB2 0SP, United Kingdom; Department of Cardiac Surgery, Royal Papworth Hospital, Papworth Road, Cambridge Biomedical Campus, Cambridge, CB2 0AY, United Kingdom; Department of Cardiac Surgery, Royal Papworth Hospital, Papworth Road, Cambridge Biomedical Campus, Cambridge, CB2 0AY, United Kingdom; Department of Cardiac Surgery, Royal Papworth Hospital, Papworth Road, Cambridge Biomedical Campus, Cambridge, CB2 0AY, United Kingdom

**Keywords:** non-bacterial thrombotic endocarditis, thrombotic neoplastic syndrome, metastatic adenocarcinoma, septic emboli, mitral valve replacement

## Abstract

A 76-year-old lady presented to her local hospital with chest pain, malaise, and fever, suspected to be due to infective endocarditis. Echocardiography showed a mass on the anterior mitral valve leaflet. Multimodal imaging showed several suspected systemic septic emboli. CT showed multiple hepatic lesions and a cavitating pulmonary lesion. Magnetic resonance imaging revealed multiple cerebral lesions. Dual antibiotic treatment was commenced, though this was unsuccessful, with persistence of her symptoms. She was therefore transferred to our tertiary centre for emergency cardiac surgery. She underwent successful bioprosthetic mitral valve replacement and initially made a good post-operative recovery. However, on the fifth post-operative day, she decompensated. Transoesophageal echocardiography showed extensive thromboses on the bioprosthetic mitral valve leaflets, in the left atrium and inferior vena cava, and on the aortic valve. Subsequently, a hepatic biopsy was performed revealing metastatic adenocarcinoma. The patient unfortunately passed away shortly after, and post-mortem examination confirmed a pulmonary adenocarcinoma with hepatic and cerebral metastases. This case highlights the importance of considering paraneoplastic processes in such cases of suspected infective endocarditis with atypical features.

## Introduction

First described in 1888, non-bacterial thrombotic endocarditis (NBTE) is characterised by sterile vegetations composed of fibrin and platelets on cardiac valves [1]. Often seen in the terminal stages of chronic diseases such as cancer, it was later known as marantic, terminal or cachectic endocarditis [2]. However, NBTE is now recognised as a potentially fatal source of systemic thromboembolism, linked with various underlying pathologies that lead to a hypercoagulable state. NBTE has been found in approximately 1.2% of patients undergoing an autopsy, with a higher prevalence in cancer patients (particularly adenocarcinomas) [3].

NBTE patients are usually asymptomatic, but around half can present with large valve vegetations mimicking infective endocarditis (IE) or with complications of systemic embolisation, highlighting the diagnostic challenge [4]. Once a diagnosis of NBTE is confirmed, the mainstay of management is controlling the underlying pathophysiology, as well as therapeutic anticoagulation (either unfractionated or low molecular weight heparin) [5]. Conversely, management of infective endocarditis includes surgery in cases of heart failure, uncontrolled infection, or established/high risk of systemic embolism [6]. We present a case of suspected infective endocarditis warranting emergent surgery, but a post-mortem diagnosis of NBTE was made after the patient deteriorated postoperatively and sadly passed away.

## Case report

A 76-year-old lady was referred to our centre for emergency mitral valve surgery. She had presented to her local hospital four days prior with chest pain, malaise, and fever, as well as nystagmus. Her only past medical history was left lower limb cellulitis, successfully treated with flucloxacillin four weeks prior.

**Figure 1:**
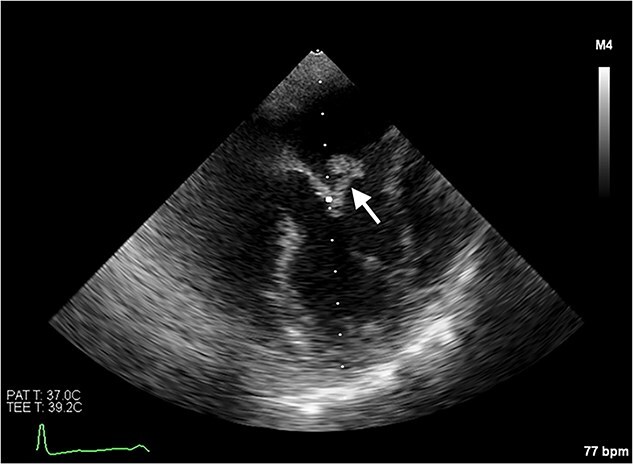
Transoesophageal echocardiogram showing the pedunculated mass on the anterior mitral valve leaflet.

**Figure 2:**
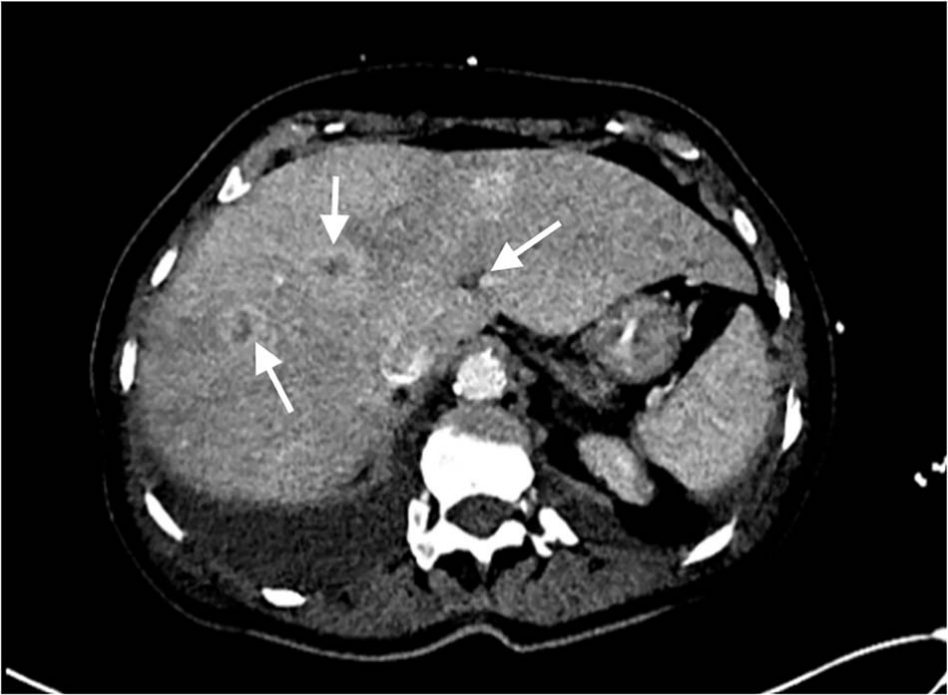
Axial CT image of the liver, showing multiple lesions, initially thought to be septic emboli.

**Figure 3:**
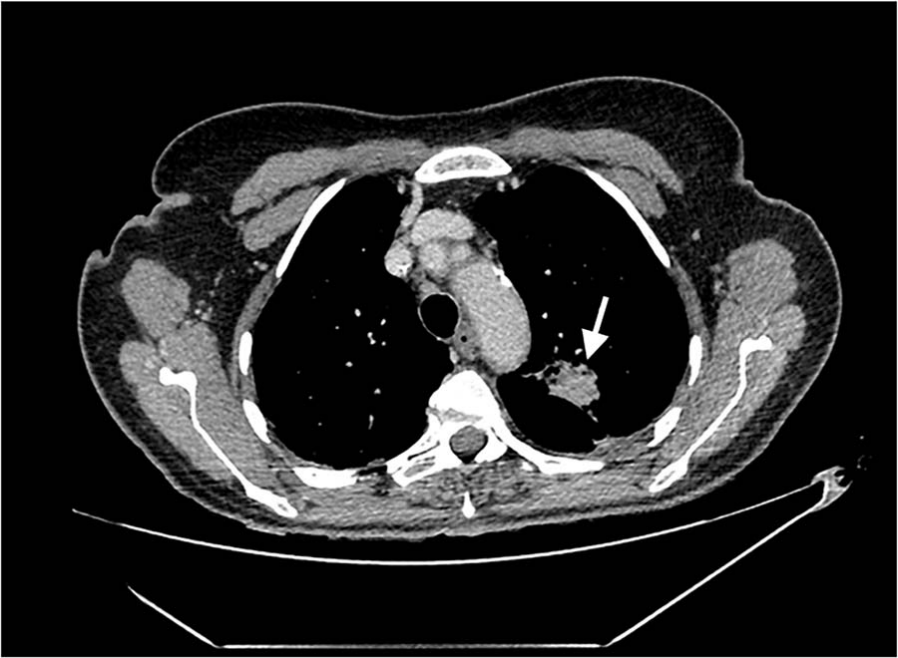
Axial CT image of the lungs, showing a cavitating mass in the posterior aspect of the left upper lobe, suggestive of an abscess secondary to infective endocarditis.

**Figure 4:**
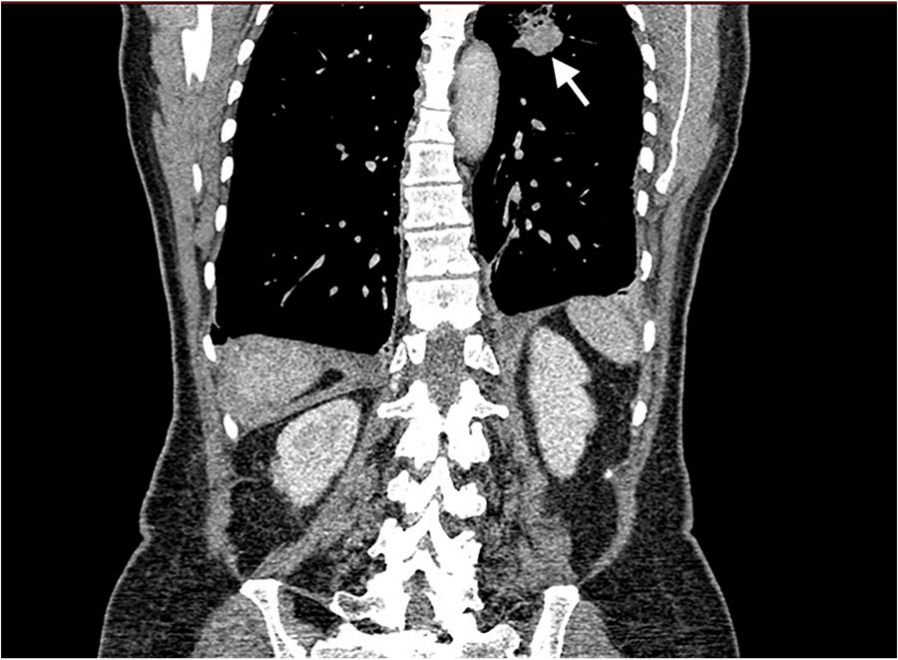
Coronal CT image of the lungs showing the cavitating mass suspected to be an abscess.

**Figure 5:**
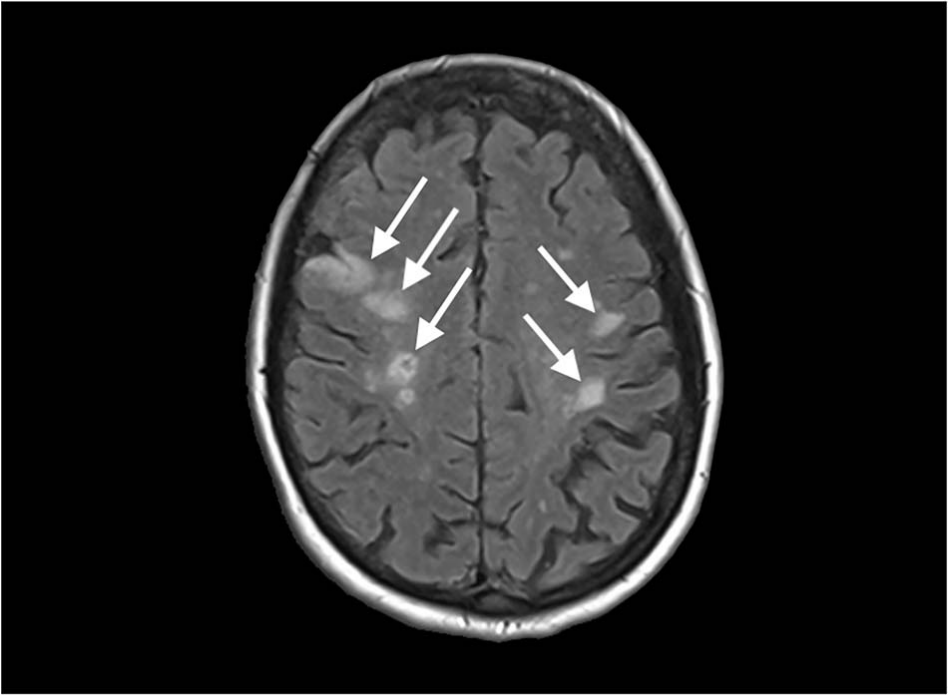
Diffusion-weighted magnetic resonance imaging image of the brain, showing multiple lesions.

Echocardiography revealed a 1.2 cm pedunculated mass attached to the anterior mitral valve leaflet (AMVL) with severe mitral regurgitation (Fig. 1). Computed tomography imaging revealed multiple hepatic lesions (suspected septic emboli, Fig. 2), and a cavitating mass in the posterior aspect of the left upper lobe of the lung (Figs 3 and 4). Magnetic resonance imaging showed multiple cerebral lesions (Fig. 5), also thought to be septic emboli. Her presentation was suspected to be due to infective endocarditis rather than ischaemic, as troponin was negative, and coronary angiography was unremarkable. She was therefore treated promptly with intravenous vancomycin and gentamicin as per local antimicrobial guidelines. However, she failed to recover, and her case was reviewed in the regional in-house urgent cardiac surgical multidisciplinary team meeting. Considering her persistent fever, as well as the recurrent emboli, the decision was made to transfer her to our centre for emergency surgery.

Following median sternotomy, cardiopulmonary bypass was instituted with bicaval drainage and ascending aortic return. Myocardial protection was achieved through antegrade delivery of warm (500 ml) followed by cold (500 ml, repeated every 20 min) blood cardioplegia into the aortic root. The mitral valve was accessed transeptally, providing excellent exposure of the annulus. A large pedunculated friable mass (1.5 cm circumference with 1 cm stalk) was identified on the atrial aspect of the AMVL (A2 scallop) and was easily detached. There was underlying damage to the anterior leaflet with vegetation extending to the annulus without evidence of peri-annular abscess.

The anterior leaflet was excised, while the posterior leaflet was preserved except for a small P2 segment showing a potential ‘kissing’ lesion. After thorough debridement, the annulus and subvalvular apparatus were soaked with vancomycin-drenched swabs for 5 min. The detached anterior papillary muscle was reattached to the anterior mitral annulus using four pledgeted 4-0 Gore-Tex neochordae. A 29 mm Hancock II bioprosthesis, pre-soaked in vancomycin-loaded saline (1 g/500 ml), was secured to the native annulus using 14 everting, pledgeted 2-0 Ethibond sutures (also pre-soaked in vancomycin solution). Transoesophageal echocardiography confirmed excellent prosthesis function with no residual regurgitation and the atrial chambers were closed with 4-0 polypropylene suture. Left ventricular function remained preserved intraoperatively. Cardiopulmonary bypass was weaned without difficulty, and the patient was transferred to the ICU in a stable cardiorespiratory condition. Emergency gram staining of the avulsed vegetation revealed no microbial growth.

**Figure 6:**
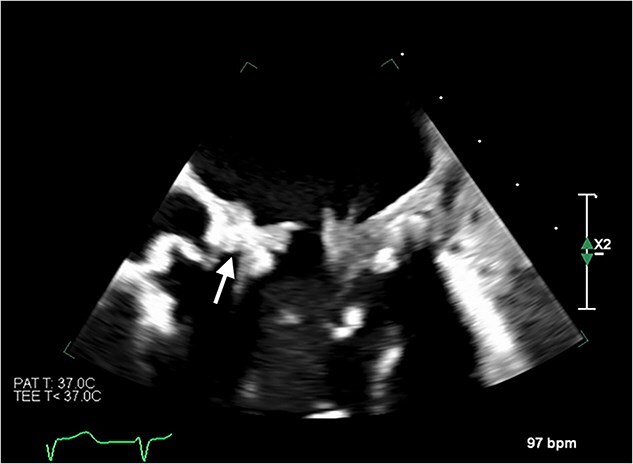
Transoesophageal echocardiogram showing thromboses on the bioprosthetic mitral valve leaflets, as well as in the left atrium and the inferior vena cava. Thrombus is also visible on the non-coronary cusp of the aortic valve.

Despite initially recovering well postoperatively, she decompensated on postoperative day 5. Blood cultures and 16S polymerase chain reaction (PCR) testing were negative. Transoesophageal echocardiography (Fig. 6) demonstrated severe bioprosthetic mitral valve dysfunction characterised by extensive thrombus on the mitral leaflets, in the left atrium, and the inferior vena cava. A small mobile mass was also observed on the aortic valve’s non-coronary cusp. Subsequent hepatic biopsy showed metastatic adenocarcinoma. The patient unfortunately passed away; post-mortem examination confirmed metastatic pulmonary adenocarcinoma with thrombotic paraneoplastic syndrome.

## Discussion

This case report presents a unique manifestation of NBTE suspected to be infective endocarditis, with subsequent rapid deterioration following emergent valve replacement surgery. The aggressive thrombotic process, masquerading as suspected infective endocarditis, highlights the diagnostic and therapeutic challenges posed by NBTE with an underlying malignancy and potential metastases. This case highlights the poor prognosis of paraneoplastic thrombotic syndrome and the futility of surgery in such cases.

We highlight the importance of maintaining a high index of suspicion for malignancy-associated thrombotic syndromes in patients presenting with atypical features of IE, such as resistance to antimicrobial therapy or the absence of microbiological evidence despite fulfilling diagnostic criteria. While the patient met multiple Duke criteria for IE (including the updated 2023 criteria) [7, 8], the absence of microbial growth, persistent embolic phenomena, and rapid recurrence of thrombotic lesions pointed to an alternative aetiology. Comprehensive diagnostic evaluation, including malignancy workup, is therefore critical in similar scenarios [3, 4].

Early recognition could prompt earlier targeted anticoagulation therapy and oncological or palliative care [5]. In such cases, surgery is unlikely to be beneficial, as valve replacement will not address the underlying pathology. When surgery is undertaken, intraoperative findings of friable, avascular vegetations should alert clinicians to the possibility of NBTE, warranting prompt investigations to elucidate the underlying pathology [1].

In this patient, the presence of multiple systemic lesions, failure of medical treatments, and rapid post-operative recurrence of thrombi were key diagnostic clues pointing to NBTE secondary to underlying metastatic malignancies. Greater clinical retrospection might have raised questions about why a patient with a left-sided cardiac lesion had resulted in what was deemed to be a pulmonary abscess due to intracardiac shunts. Such consideration could have facilitated an earlier diagnosis and more appropriate treatment. This case underscores the difficulty in differentiating between infectious and such paraneoplastic processes, highlighting the importance of comprehensive clinical evaluation in presentations of suspected infective endocarditis with atypical features.

## References

[1] Lopez JA, Ross RS, Fishbein MC, et al. Nonbacterial thrombotic endocarditis: a review. Am Heart J 1987;113:773–84. 10.1016/0002-8703(87)90719-8 3548296

[2] Zmaili M, Alzubi J, Lo Presti Vega S, et al. Non-bacterial thrombotic endocarditis: a state-of-the-art contemporary review. Prog Cardiovasc Dis 2022;74:99–110. 10.1016/j.pcad.2022.10.009 36279942

[3] Asopa S, Patel A, Khan OA, et al. Non-bacterial thrombotic endocarditis. Eur J Cardiothorac Surg 2007;32:696–701. 10.1016/j.ejcts.2007.07.029 17881239

[4] Rose D, Sissons M, Zacharias J. A large aortic valve vegetation: an unusual case of non-bacterial thrombotic (marantic) endocarditis. Eur J Cardiothorac Surg 2017;51:603–4. 10.1093/ejcts/ezw334 28364443

[5] Whitlock RP, Sun JC, Fremes SE, et al. Antithrombotic and thrombolytic therapy for Valvular disease: antithrombotic therapy and prevention of thrombosis, 9th ed: American College of Chest Physicians Evidence-Based Clinical Practice Guidelines. Chest 2012;141:e576S–600S. 10.1378/chest.11-2305 22315272 PMC3278057

[6] Delgado V, Ajmone Marsan N, De WS, et al. 2023 ESC guidelines for the management of endocarditis: developed by the task force on the management of endocarditis of the European Society of Cardiology (ESC) endorsed by the European Association for Cardio-Thoracic Surgery (EACTS) and the European Association of Nuclear Medicine (EANM). Eur Heart J 2023;44:3948–4042. 10.1093/eurheartj/ehad193 37622656

[7] Durack DT, Lukes AS, Bright DK. New criteria for diagnosis of infective endocarditis: utilization of specific echocardiographic findings. Duke Endocarditis Service Am J Med 1994;96:200–9. 10.1016/0002-9343(94)90143-0 8154507

[8] Fowler VG, Durack DT, Selton-Suty C, et al. The 2023 Duke-International Society for Cardiovascular Infectious Diseases criteria for infective endocarditis: updating the modified Duke criteria. Clin Infect Dis 2023;77:518–26. 10.1093/cid/ciad271 37138445 PMC10681650

